# Sex-Specific Differences and the Role of Environmental Enrichment in the Expression of Hippocampal CB_1_ Receptors following Chronic Unpredictable Stress

**DOI:** 10.3390/brainsci14040357

**Published:** 2024-04-03

**Authors:** Evgenia Dandi, Evangelia Kesidou, Constantina Simeonidou, Evangelia Spandou, Nikolaos Grigoriadis, Despina A. Tata

**Affiliations:** 1 Laboratory of Cognitive Neuroscience, School of Psychology, Aristotle University of Thessaloniki, 54124 Thessaloniki, Greece; dandi.evgenia@gmail.com; 2 Laboratory of Experimental Neurology and Neuroimmunology, 2nd Department of Neurology, AHEPA University Hospital, Aristotle University of Thessaloniki, 54636 Thessaloniki, Greece; kesidoue@auth.gr (E.K.); ngrigoriadis@auth.gr (N.G.); 3 Laboratory of Physiology, School of Medicine, Aristotle University of Thessaloniki, 54124 Thessaloniki, Greece; ksymeoni@auth.gr (C.S.); espandou@auth.gr (E.S.)

**Keywords:** chronic unpredictable stress (CUS), endocannabinoids (eCBs), hippocampus, sex differences, enriched environment (EE)

## Abstract

Stress-related mental disorders have become increasingly prevalent, thus endangering mental health worldwide. Exploring stress-associated brain alterations is vital for understanding the possible neurobiological mechanisms underlying these changes. Based on existing evidence, the brain endogenous cannabinoid system (ECS) plays a significant role in the stress response, and disruptions in its function are associated with the neurobiology of various stress-related disorders. This study primarily focuses on investigating the impact of chronic unpredictable stress (CUS) on the expression of hippocampal cannabinoid type 1 (CB_1_) receptors, part of the ECS, in adult male and female Wistar rats. Additionally, it explores whether environmental enrichment (EE) initiated during adolescence could mitigate the CUS-associated alterations in CB_1_ expression. Wistar rats, shortly after weaning, were placed in either standard housing (SH) or EE conditions for a duration of 10 weeks. On postnatal day 66, specific subgroups of SH or EE animals underwent a 4-week CUS protocol. Western blot (WB) analysis was conducted in the whole hippocampus of the left brain hemisphere to assess total CB_1_ protein expression, while immunohistochemistry (IHC) was performed on the right hemisphere to estimate the expression of CB_1_ receptors in certain hippocampal areas (i.e., CA1, CA3 and dentate gyrus-DG). The WB analysis revealed no statistically significant differences in total CB_1_ protein levels among the groups; however, reduced CB_1_ expression was found in specific hippocampal sub-regions using IHC. Specifically, CUS significantly decreased CB_1_ receptor expression in the CA1 and DG of both sexes, whereas in CA3 the CUS-associated decrease was limited to SH males. Interestingly, EE housing proved protective against these reductions. These findings suggest a region and sex-specific endocannabinoid response to chronic stress, emphasizing the role of positive early experiences in the protection of the adolescent brain against adverse conditions later in life.

## 1. Introduction

Chronic stress is implicated in the onset of various psychological disorders as well as neurodegenerative diseases caused by increased cortisol production and subsequent hypothalamic–pituitary–adrenal (HPA) axis dysregulation [1,2,3,4,5]. To better understand the effects and pathophysiology of stressful experiences, various animal paradigms have been introduced, including the Chronic Unpredictable Stress protocol, which mimics stress experienced in humans [6,7]. By studying the multifaceted stress mechanisms, scientists aim to identify potential interventions to mitigate the detrimental effects of chronic stress. The endogenous cannabinoid system (ECS) is recognized as a key component in the stress response, regulating HPA axis activity [8]. Moreover, ECS plays a significant role in the central nervous system (CNS) by modulating various brain functions, such as the reward behavior, pain perception, and learning and memory [9,10,11]. The endogenous molecules, known as endocannabinoids (eCBs), inhibit norepinephrine release [12] and their action is mediated by two types of receptors, cannabinoid type 1 and type 2 (CB_1_, CB_2_). CB_2_ receptors, expressed mainly in the peripheral immune system, exert an immunomodulatory effect, with CB_2_ agonists proposed as a potential treatment for various conditions such as chronic inflammatory pain, multiple sclerosis, and inflammatory bowel disease, among others [13,14]. In contrast, CB_1_ receptors, the focus of the current study, are expressed mostly in the CNS, modulating synaptic plasticity as well as behavioral, autonomic and somatic functions [15]. 

In fact, activation of CB_1_ receptors produces anxiolytic effects in various animal models of stress [16]. Studies involving CB_1_ knockout mice reveal aggressive responses and depressive-like behavior, indicating the involvement of endocannabinoids (eCBs) through the activation of CB_1_ receptors, in the regulation of emotional behavior [17]. The hippocampus, amygdala and prefrontal cortex are some of the brain regions rich in CB_1_ receptors, also participating in HPA axis negative feedback [18]. Under acute stress, glucocorticoids trigger the ECS, leading to the activation of CB_1_ receptors, downregulating HPA axis activity [19]. Conversely, chronic exogenous corticosterone (CORT) administration and/or exposure to chronic stress decreases hippocampal CB_1_ receptor signaling, causing dysregulation of the HPA axis activity due to increased levels of circulating CORT [20,21,22]. Despite the significance of the ECS in managing the stress response [8] and the investigation of eCBs as a potential therapeutic target for both preventing and managing stress-related mental disorders [16,23], the current understanding in this field is limited.

Existing evidence suggests that the regulation of CB_1_ receptors in response to CUS may be region and sex specific. In the CUS paradigm, animals are exposed daily to a variety of stressors over a specific period of time. The variety of stressors and the unpredictability in the timing and sequence of stressor administration to prevent habituation render CUS paradigm an ethologically relevant model for studying the effects of psychological stress in humans [24,25,26,27]. Research findings from studies in which CUS was implemented indicate a reduction in CB_1_ receptor expression in the hippocampus, while increased expression is observed in the amygdala and prefrontal cortex (PFC) [28,29]. The diminished expression of CB_1_ receptors in the hippocampus has been linked to anxiety and depressive-related behavior [30], while the increased activation of CB_1_ receptors in the amygdala and PFC prevents CORT elevations in response to stress [31]. Recent evidence-based research has underscored the significance of including both sexes for direct comparison. However, only a restricted amount of research has explored the potential positive impacts of EE against chronic stress in both sexes simultaneously [27]. Interestingly, studies encompassing both sexes have demonstrated that CUS decreases hippocampal CB_1_ expression in males, whereas it increases their expression in females [32,33]. In other brain areas, such as the PFC, CB_1_ receptor expression increases in response to CUS, but no sex differences have been identified [32]. Differences in CB_1_ receptor expression are not solely confined to stress-related conditions; data indicate that disparities may also be evident in naive males and females, and these differences appear to be influenced by factors such as sex hormones, brain region, and the developmental stage [34]. 

Pharmacological agents modulating the ECS have demonstrated efficacy in alleviating depressive and anxiety disorders as well as conditions such as post-traumatic stress disorder (PTSD, attention deficit–hyperactivity disorder (ADHD), Tourette syndrome, and psychosis [35]. Existing evidence indicates that the ECS acts as a homeostatic mechanism preventing unnecessary activation of the HPA axis and restoring its activity to baseline following stress [36]. The eCBs and their target receptors CB_1_ play a critical role in the brain’s adaptation to repeated stress. Consequently, the eCB signaling system is being studied as a potential therapeutic target for both preventing and treating stress-related psychopathology [16,23]. 

The enriched environment (EE), described as “a new way of endogenous pharmacotherapy” [37], acts protectively against the negative effects induced by stressful experiences [38,39,40] and exerts significant molecular, anatomical and functional changes in the brain [41]. In animal studies, the EE condition refers to the housing of more than two same-sex animals in large cages equipped with various objects of varying shapes and sizes along with running wheels. This environmental manipulation offers cognitive and sensory stimulation while encouraging exploration and social interaction among animals [27,42]. Numerous studies have demonstrated that EE delays the onset and progression of psychiatric disorders and neurological diseases in different animal models [43]. Early exposure to EE induces alterations in the ECS in brain regions pivotal to the stress response. In line with the above, El Rawas and colleagues found that EE led to an increase in CB_1_ mRNA levels in the hypothalamus and basolateral amygdala while decreasing them in basomedial amygdala. These EE-associated alterations have been implicated in the reduction in stress-related behavior [44]. In general, the increases in CB_1_ expression and activation in the brain as a result of EE exposure have been associated with reduced anxiety [37,45]. While the involvement of CB_1_ receptors in the stress response has been well documented [20,21,22] and EE has been shown to increase their expression [44], there is currently a lack of evidence concerning the beneficial effects of EE in the endogenous cannabinoid system against the negative effects caused by CUS. 

To date, there have only been a limited number of studies examining the interaction between environmental enrichment (EE) and chronic unpredictable stress (CUS), with most of these investigations focusing primarily on EE–CUS interaction in male animals. The aim of the present study is to explore if housing in enriched environmental conditions during adolescence and adulthood could act protectively against the CUS-associated changes in CB_1_ expression in the hippocampus. It was hypothesized that animals exposed to CUS would exhibit significant decreases in the expression of CB_1_ receptors in the hippocampus, while EE manipulation would reverse this effect. Acknowledging the modulatory role of eCBs in the stress response and considering the importance of sex in stress-associated disorders, Wistar rats of both sexes were included in our study. To the best of our knowledge, this is the first study to examine the impact of EE, initiated in adolescence concurrently in female and male rats subjected to CUS in adulthood, on CB_1_ receptor expression in three distinct subregions of the hippocampus as well as in the hippocampus as a whole. 

## 2. Materials and Methods

### 2.1. Animals 

Wistar rats were obtained from the Veterinary Medicine School of Aristotle University of Thessaloniki. Rats originated from 12 litters (litter size: 5–10 rats) and were maintained in a controlled environment with a 12 h light/12 h dark cycle (lights on at 08:00/lights off at 20:00) at a standard temperature of 22 ± 2 °C, and they had unrestricted access to food and water. All experimental procedures were complied with the European Communities Council Directive (2010/63/EU) on the protection of animals, and the experimentation protocol was approved by the local Veterinary Medicines Directorate (#471643-1811). The sample size was calculated using the G*Power software (version 3.1.9.7) with a power of 0.8, an alpha error of 0.05 and Wilcoxon–Mann–Whitney tests.

### 2.2. Experimental Manipulations 

On postnatal day 24 (PND24), animals were randomly housed either in standard laboratory (SH) cages (42.5 cm × 26.6 cm × 18.5 cm) or in enriched environment (EE) cages (76 cm × 45 cm × 60 cm) for a period of 10 weeks, until the termination of the experimental manipulations (PND 107). Five same-sex rats were housed in EE cages equipped with running wheels, non-chewable toys, ladders and platforms. On PND 66, a subset from SH and EE groups underwent daily exposure to a Chronic Unpredictable Stress (CUS) protocol for four weeks (PND66-95), while the other remained undisturbed (No Stress/NS) in a separate colony room. The resulting groups were as follows: Standard Housing/No Stress (SH/NS); Enriched Environment/No Stress (EE/NS); Standard housing/Chronic Unpredictable Stress (SH/CUS); Enriched Environment/Chronic Unpredictable Stress (EE/CUS). The CUS protocol included a variety of physiological, psychological, and social stressors over a four-week period. The CUS-exposed rats were subjected to two different stressors per day (light and/or dark phases), while the NS rats received 1 min handling every two days to compensate for the handling experienced by the stressed groups (for a detailed description of the protocol, please refer to [38]).

### 2.3. Tissue Processing 

**Figure 1:**
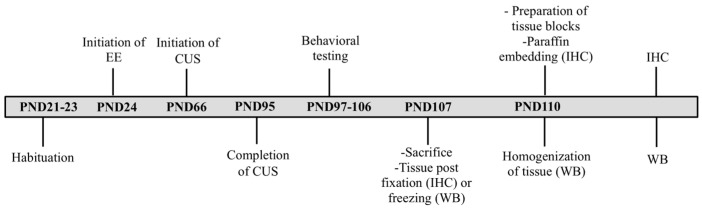
Schematic representation of the timeline and experimental conditions of the study. EE = environmental enrichment; CUS = chronic unpredictable stress; IHC = immunohistochemistry; WB = Western blotting.

On PND 107, the animals were euthanized after being anesthetized with an intraperitoneal injection of ketamine (40 mg/kg) and xylazine (3 mg/kg). Prior to euthanasia (PND 99-106), behavioral testing was conducted to explore cognitive and emotional behavior The battery included the Barnes Maze (spatial learning and memory), the Elevated Plus Maze (anxiety), the Forced Swimming Test (depressive-like behavior) and the Open Field Test (anxiety, ambulatory activity) [38]. To assess total CB_1_ protein expression, Western blot (WB) analysis was conducted in the whole hippocampus of the left brain hemisphere, while immunohistochemistry (IHC) was performed on the right hemisphere to estimate the expression of CB_1_ receptors in certain hippocampal areas (i.e., CA1, CA3 and dentate gyrus-DG). For WB analysis, snap frozen left hippocampi were homogenized in ice-cold lysis buffer (containing 10 mM Hepes pH 7.4, 10 mM KCL, 0.1 mM EDTA, 0.1 mM EGTA, 1 mM DTT and a mixture of protease inhibitors). For IHC analysis, the right hemispheres were promptly post-fixed in 4% paraformaldehyde (3 × 24 h at 4 °C) and following preparation of tissue blocks, tissue underwent gradual hydration before being paraffin embedded. Subsequently, coronal sections (5 μm) were obtained through the dorsal hippocampus (corresponding to coronal coordinates −3.24 to −3.36 mm from bregma [46]). Figure 1 illustrates the experimental design of the study.

#### 2.3.1. Immunohistochemical Analysis

CB_1_ receptor expression was estimated in the right hemisphere brain tissue of 40 animals as follows: SH/NS (*n* = 5 males, *n* = 5 females), SH/CUS (*n* = 5 males, *n* = 5 females), EE/NS (*n* = 5 males, *n* = 5 females), EE/CUS (*n* = 5 males, *n* = 5 females), using immunohistochemistry and light microscopy. In order to maintain consistent labeling conditions, tissue was collected on the same day, stored under identical conditions, and processed in parallel batches. Following deparaffinization and hydration, sections were treated with 3% hydrogen peroxide (H_2_O_2_)/methanol (10 min). Antigen retrieval was performed in citrate buffer (0.1 M, pH 6.0), followed by rinsing with phosphate-buffered saline (PBS) and incubation (1.5 h) in blocking buffer (10% fetal bovine sodium, 2% normal goat serum). Sections were exposed overnight (4 °C) to a primary antibody against CB_1_ receptor (Abcam, Cambridge, UK, rabbit polyclonal, 1:100), followed by incubation in a secondary antibody (Goat anti-rabbit, 1:200, 1.5 h at room temperature). Hematoxylin served as the nuclear counterstaining agent, and 3,3′ diaminobenzidine (DAB; Vector Laboratories, Newark, CA, USA) was used as the chromogen to visualize immunoreaction for CB_1_ receptors. Sections treated with the same procedure but in the absence of the CB_1_ antibody exhibited no positive immunostaining and served as negative controls. 

Images of hippocampal tissue were obtained with a digital camera (Nikon DS–5M–L1) connected to a microscope (Nikon Eclipse 50i, Tokyo, Japan). The CB_1_ receptor immunoreactivity was assessed in the dentate gyrus (DG) molecular layer and CA3 and CA1 stratum radiatum. Two sections were selected per animal, and in each section, three IHC images per subregion (DG, CA3, CA1) were obtained. The DAB signal was blindly quantified and analyzed using the ImageJ/Fiji software (version 1.2). The mean percentage of CB_1_-positive tissue area per hippocampal region was calculated as an indicator of CB_1_ immunoreactivity (for more details, see [39]). 

#### 2.3.2. Western Blot (WB) Analysis 

The CB_1_ protein levels in the hippocampus of 40 animals (SH/NS, *n* = 5 males, *n* = 5 females; SH/CUS, *n* = 5 males, *n* = 5 females; EE/NS, *n* = 5 males, *n* = 5 females; EE/CUS, *n* = 5 males, *n* = 5 females) were evaluated using WB. Following homogenization of snap-frozen samples in ice-cold lysis buffer, the protein concentration was estimated using a DC protein assay kit (Biorad, Hercules, CA, USA). Total protein lysate (20–30 μg) was separated using SDS-PAGE and transferred onto polyvinylidene fluoride (PVDF) membranes (Macherey-Nagel, GmbH&Co, Düsseldorf, Germany). Protein was probed with an anti-CB1 receptor antibody (Abcam, rabbit polyclonal, 1:500), followed by washes of membranes with PBS supplemented with 0.1% Tween 20 (PBST) and incubation with HRP-conjugated goat anti-rabbit IgG antibody (Sigma-Aldrich, St. Louis, MO, USA, 1:10,000). The immunoreactivity was visualized using enhanced chemiluminescence (ECL, GenScript, Piscataway, NJ, USA). Subsequently, all membranes were stripped and re-probed with an anti-actin antibody (Cell Signaling Technology, Leiden, The Netherlands) as a loading control. The immunoblot signal was normalized to actin, and densitometric analysis was blindly performed using Image J/Fiji software.

### 2.4. Statistical Analysis

The main effects of stress, housing and sex and their interactions on CB_1_ immunoreactivity and total CB_1_ protein were explored with 2 (stress: NS, CUS) × 2 (type of housing: SH, EE) × 2 (sex: males, females) analyses of variance (ANOVAs). The statistical program SPSS Statistics (v. 27) was used. Data satisfied the requirements for normality. To investigate statistically significant interactions between CUS and EE, simple effects tests were employed to assess the impact of one factor at each level of other factors. These analyses were conducted separately in each sex group to further examine potential statistically significant differences between males and females that might be obscured if they have been analyzed together [47]. Simple effects tests were performed in each sex group using the COMPARE subcommand in the SYNTAX editor of SPSS [48]. Data are displayed as mean values ± standard error of the mean (SEM) and are graphed according to sex. For all analyses, statistical significance was established at *p* < 0.05.

## 3. Results

### 3.1. Analysis of CB_1_ Expression in DG, CA3 and CA1 by Immunohistochemistry 

#### 3.1.1. EE Exhibited a Protective Effect against the Stress-Induced Reductions in the Percentage of CB_1_-Positive Area in DG

**Figure 2:**
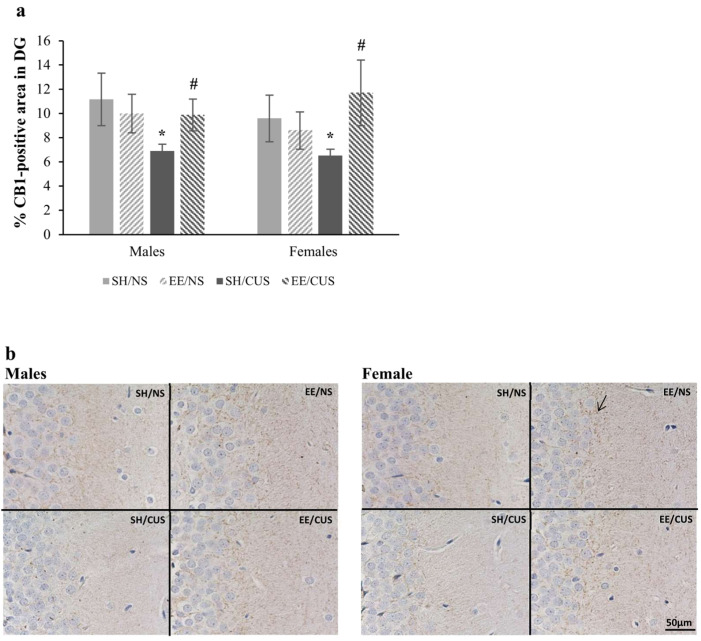
Expression of CB_1_ receptors in DG molecular layer. (**a**) CUS significantly reduced the CB_1_ receptor immunoreactivity for SH rats (SH/NS vs. SH/CUS, * *p* < 0.05). Stressed rats that were exposed to EE did not exhibit this decrease (SH/CUS vs. EE/CUS, ^#^ *p* < 0.05). (**b**) Representative photomicrographs from the DG exposed to CB_1_ antibody (DAB visualization). The arrow indicates positive immunostaining for CB_1_ receptors. Total magnification 400×, scale bar = 50 μm; *n* = 5 per group. SH = standard housing; EE = environmental enrichment; NS = no stress; CUS = chronic unpredictable stress.

The analysis of CB_1_ immunoreactivity in the DG molecular layer revealed a significant interaction between stress and housing [F(1,32) = 4.65, *p* = 0.039, partial η^2^ = 0.127]. Simple effects analysis demonstrated a significant reduction in the CB_1_-positive area induced by stress in SH animals [F(1,32) = 4.68, *p* = 0.038, partial η^2^ = 0.128; SH/NS = 10.4 ± 1.4 vs. CUS/SH = 6.7 ± 0.4]. Interestingly, EE reversed this stress-associated decrease [F(1,32) = 5.81, *p* = 0.022, partial η^2^ = 0.154; SH/CUS = 6.7 ± 0.4 vs. EE/CUS = 10.8 ± 1.5] (Figure 2).

#### 3.1.2. EE Exhibited a Protective Effect against the Stress-Induced Reductions in the Percentage of CB_1_-Positive Area in CA3 of Male Rats

**Figure 3:**
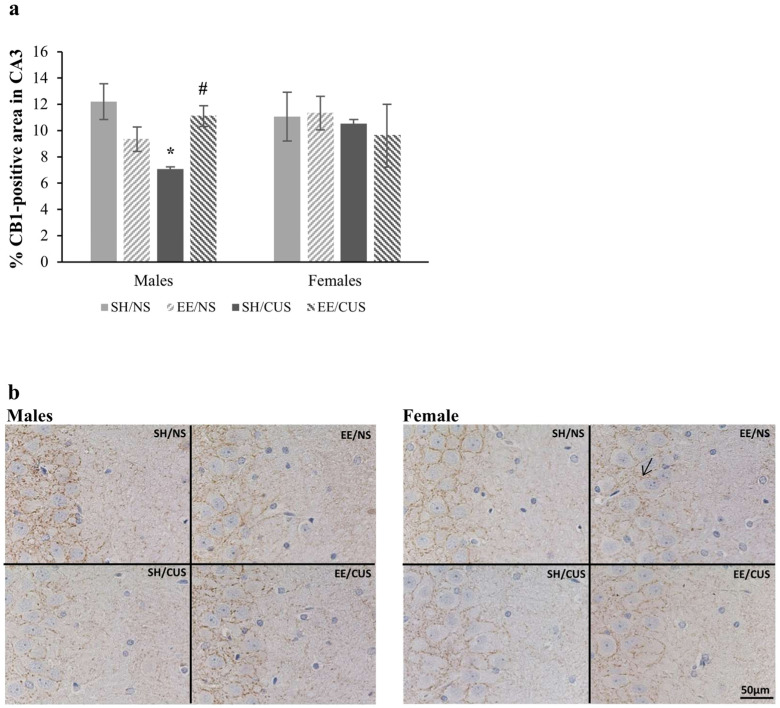
Expression of CB_1_ receptors in the CA3 stratum radiatum. (**a**) CUS significantly decreased the CB_1_ receptor immunoreactivity in SH male rats only (SH/NS vs. SH/CUS, * *p* < 0.05). The EE condition reversed this reduction (SH/CUS vs. EE/CUS, ^#^ *p* < 0.05). (**b**) Representative photomicrographs from the CA3 exposed to CB_1_ antibody (DAB visualization). The arrow indicates positive immunostaining for CB_1_ receptors. Total magnification 400×, scale bar = 50 μm; *n* = 5 per group. SH = standard housing; EE = environmental enrichment; NS = no stress; CUS = chronic unpredictable stress.

The statistical analysis of the CB_1_-positive area in the CA3 stratum radiatum showed a significant interaction among stress, housing, and sex [F(1,32) = 4.58, *p* = 0.04, partial η^2^ = 0.125]. According to the analysis of simple effects, the expression of CB_1_ receptors was significantly reduced only in SH/CUS males [F(1,32) = 7.41, *p* = 0.01, partial η^2^ = 0.188; SH/NS = 12.2 ± 1.9 vs. SH/CUS = 7.1 ± 0.3]. Animals housed in EE conditions did not exhibit this CUS-associated decrease [F(1,32) = 4.59, *p* = 0.04, partial η^2^ = 0.125; SH/CUS = 7.1 ± 0.3 vs. EE/CUS = 11.1 ± 2.4] (Figure 3). 

#### 3.1.3. EE Exhibited a Protective Effect against the Stress-Induced Reductions in the Percentage of CB_1_-Positive Area in CA1

**Figure 4:**
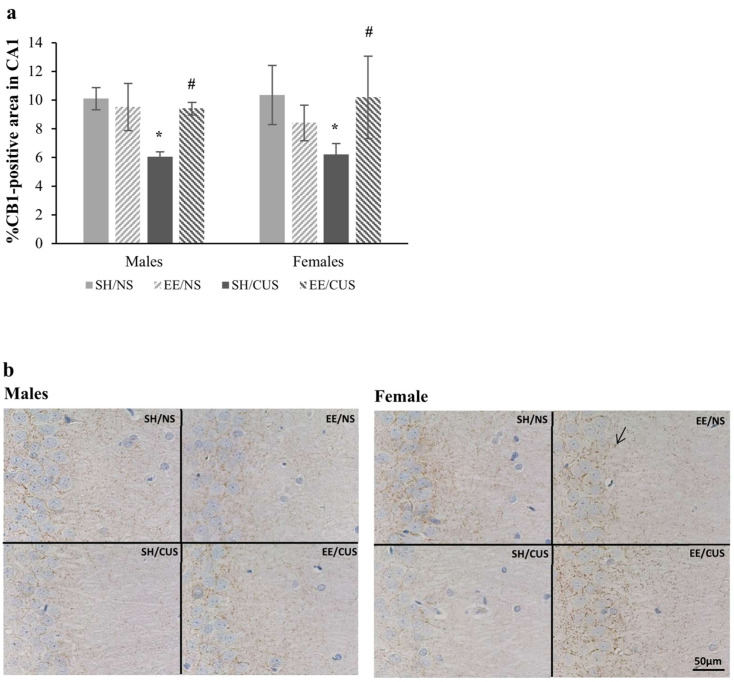
Expression of CB_1_ receptors in the CA1 stratum radiatum. (**a**) CUS significantly decreased the CB_1_ receptor immunoreactivity in SH rats (SH/NS vs. SH/CUS, * *p* < 0.05). The EE condition reversed this effect (SH/CUS vs. EE/CUS, ^#^ *p* < 0.05). (**b**) Representative photomicrographs from the CA1 exposed to CB_1_ antibody (DAB visualization). The arrow indicates positive immunostaining for CB_1_ receptors. Total magnification 400×, scale bar = 50 μm; *n* = 5 per group. SH = standard housing; EE = environmental enrichment; NS = no stress; CUS = chronic unpredictable stress.

The analysis of CB_1_ immunoreactivity in the CA1 stratum radiatum revealed a significant interaction between stress and housing conditions [F(1,32) = 5.33, *p* = 0.03, partial η^2^ = 0.143]. Further examination through simple effects analysis demonstrated that CUS significantly decreased the expression of CB_1_ receptors in standardly housed rats [F(1,32) = 7.36, *p* = 0.011, partial η^2^ = 0.187; SH/NS = 10.2 ± 1 vs. SH/CUS = 6.1 ± 0.4]. EE housing reversed this outcome [F(1,32) = 5.89, *p* = 0.021, partial η^2^ = 0.155; SH/CUS = 6.1 ± 0.4 vs. EE/CUS = 9.8 ± 1.4] (Figure 4).

### 3.2. Analysis of CB_1_ Total Protein Expression in the Hippocampus Using Western Blotting

**Figure 5:**
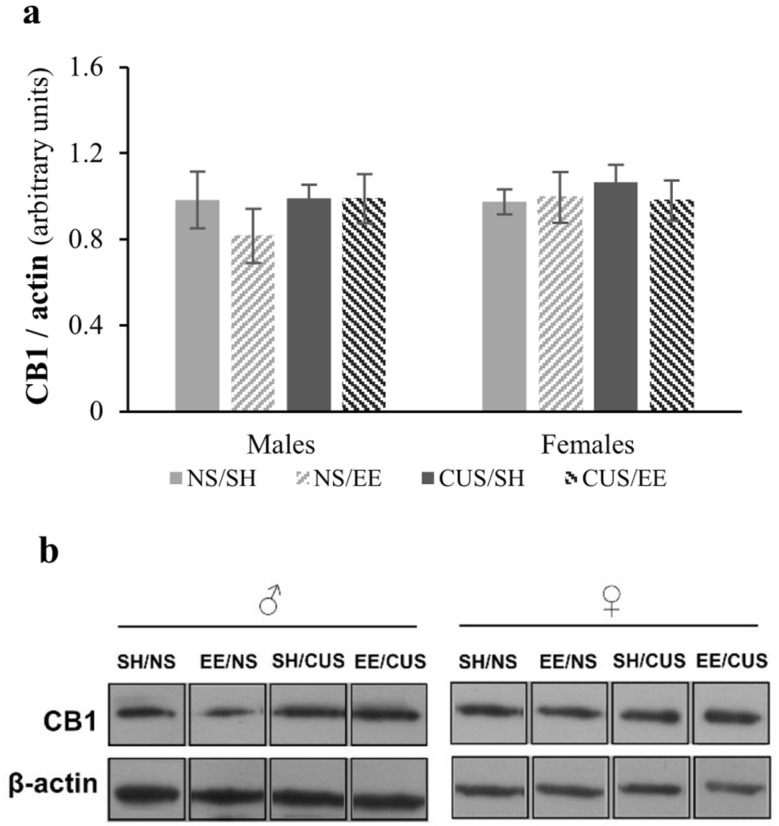
Total CB_1_ hippocampal protein expression. (**a**) No significant differences were found among groups (*p* > 0.05). (**b**) Representative Western blots are shown below the graph, representing the CB_1_ band. The immunoblot signal of CB_1_ was normalized to actin. *n* = 5 per group. SH = standard housing; EE = environmental enrichment; NS = no stress; CUS = chronic unpredictable stress.

Statistical analysis of the CB_1_ total protein expression showed no significant main effect of stress [F(1,32) = 0.79, *p* = 0.38, partial η^2^ = 0.024], housing [F(1,32) = 0.64, *p* = 0.43, partial η^2^ = 0.020] or sex [F(1,32) = 0.66, *p* = 0.42, partial η^2^ = 0.020]. The factors did not interact with each other [stress × housing × sex: F(1,32) = 0.89, *p* = 0.35, partial η^2^ = 0.027] (Figure 5). 

## 4. Discussion

Existing evidence indicates that stress-induced disruptions in the brain ECS signaling might be involved in the neurobiology of stress-related disorders such as PTSD and depression. Thus, the ECS is regarded as a potential target system for drug development in psychiatric disorders related to stress [49]. Previous studies have demonstrated that CB_1_ receptor inhibition increases the symptoms of depression and anxiety [50,51], even in individuals without pre-existing conditions [52]. On the contrary, the administration of CB_1_ receptors agonists exerts antidepressant effect [51]. The aim of the present investigation was to explore the impact of CUS and EE on the expression of CB_1_ receptors in the hippocampus of adult male and female Wistar rats. To date, relatively few investigations on EE–CUS interaction have been carried out, with the majority of these studies mainly employing male animals (for a review, see [27]). 

Our findings suggest that the statistically significant reduction in the expression of CB_1_ receptors in distinct hippocampal areas following adult CUS is both sex and region specific. As WB analysis revealed, no significant difference in total CB_1_ protein levels was found. However, IHC analysis showed sex-related decreases in CB_1_ receptor expression in specific hippocampal subregions. Specifically, CUS led to a significant reduction in CB_1_ receptor expression in the CA1 and DG for both sexes, whereas in CA3 the decrease associated with CUS was observed only in SH males. Prior research has reported decreased levels of hippocampal CB_1_ receptor expression in CUS adult male rats [30]. Conversely, our findings differ from other studies that support an increase in hippocampal CB_1_ receptor expression in females subjected to CUS [32,33].

Determining the regional regulation of hippocampal CB_1_ receptor expression following stress is essential for understanding the functional connection between stress-induced CB_1_ receptor signaling and neuroplasticity [31]. Most of the existing studies focus on the effects of stress on the CB_1_ receptor in the entire hippocampus. However, our results suggest that this approach might mask some of the region-specific effects of stress. Hill and colleagues reported decreases in CB_1_ receptor binding in the DG, increases in the CA3 region, and no difference in CA1 in male rats after 21 days of restraint stress [31]. One potential explanation for the divergent results in relation to the present study could be attributed to the type of chronic stress employed in previous investigations. Specifically, chronic exposure to homotypic stress is less likely to increase CORT levels, and the alterations noted in CB_1_ receptor expression are less robust compared to those observed following CUS [21]. Additionally, sex hormones and developmental stages may contribute to these differences (for a review, see [34]). For instance, CB_1_ receptor expression is higher in CA3 and DG in adolescent male rats compared to adult male rats [53].

Despite the pivotal role of the ECS in the stress response [8], and the exploration of eCBs as a potential therapeutic target for both preventing and treating stress-related psychopathology [16,23], the current state of knowledge in this area is limited. To date, research has mainly focused on investigating the therapeutic role of the ECS in various conditions such as chronic pain, epilepsy, neurodegenerative diseases, and cancer, among others. In addition, the majority of studies have employed pharmacological interventions to modulate eCBs for the improvement of behavior and diseases [54]. For instance, it has been demonstrated that the activation of the ventral hippocampal CB_1_ receptors by cannabinoid receptor agonists can inhibit the anxiogenic-like behaviors induced by ketamine [55]. 

In the present study, we opted for EE as a non-pharmacological intervention to investigate its protective role against the CUS-associated changes in CB_1_ expression in the hippocampus. Our results indicate that EE housing, initiated in adolescence, prevented the reduction in CB_1_ receptor expression in all three hippocampal subregions caused by CUS. Existing evidence supports the positive impact of EE on both brain and behavior [41,56], demonstrating its efficacy in mitigating deficits associated with various brain-related disorders or exposure to adverse conditions throughout life [40,57]. While studies support that EE increases CB_1_ expression and activation in the brain and that these alterations are associated with reduced anxiety [37,45], there is currently a lack of evidence concerning the beneficial impact of EE on alterations in the endogenous cannabinoid system caused by CUS. To our knowledge, the current study is the first to explore the potential interaction between EE, initiated during adolescence, and CUS in adulthood, on the expression of CB_1_ receptors in three distinct subregions of the hippocampus in both male and female rodents.

Based on our findings, the adverse effects of CUS on cognitive function and emotional behavior [38], which were also linked to a region and sex-specific decrease in the expression of glial fibrillary acidic protein (GFAP) and synaptophysin (SYN) in the hippocampus [39], may be mediated by alterations in the CB_1_ receptor expression. Specifically, the learning impairments previously found exclusively in CUS males could be associated with the decreases found in SYN and CB_1_ receptor expression in the CA3 and CA1 hippocampal areas. Meanwhile, the decreases in SYN and CB_1_ receptor expression in CA1 observed in females may explain the increase in depressive behavior detected only in females [38,39]. In support of the present findings, existing evidence highlights the role of eCBs in modulating synaptic function and contributing to synaptic plasticity through retrograde signaling to CB_1_ and CB_2_ receptors [58]. Chronic exposure to repeated restraint or foot shock stress has been shown to alter CB_1_ receptor signaling, leading to deficits in learning and memory, as well as to dysfunctional behavioral responses to stress [59,60].

## 5. Conclusions

Our findings support the potential role of ECS signaling in the brain in response to chronic stress. The regional altered expression of CB_1_ receptors in animals exposed to CUS indicates that ECS signaling might have a region-specific regulatory role in synaptic function, while factors such as sex hormones may be associated with the sex-dependent changes. The reversal of CUS-associated reduction in the expression of CB_1_ receptors with EE housing emphasizes the positive role of this environmental manipulation against the detrimental effects of CUS.

Preclinical studies provide valuable insights into understanding organism function and in the development of suitable treatments, but it is crucial to interpret their findings with caution when considering clinical applications. The current study may lay the foundation for further research regarding the protective effect of enriched environmental experiences against the adverse impact of chronic stress on both behavior and the brain. The investigation of the significance of sex/gender in these effects is of great importance. The findings of the current study underscore the importance of early-life prevention, suggesting that children and adolescents raised in environments that promote cognitive flexibility and social interactions are inclined to be more resilient to stressful experiences as adults. Although the beneficial effects of EE on behavior and the brain are well-documented and existing evidence supports its beneficial role in stressful conditions, there are still important aspects that require further investigation. Future investigations should not only take into account both sexes but should also incorporate analyses of further alterations in structure and function in additional stress-associated brain regions such as the prefrontal cortex and the amygdala.

## Data Availability

Dataset available on request from the authors.
